# 异基因造血干细胞移植治疗急性髓系白血病伴骨髓增生异常相关改变75例临床分析

**DOI:** 10.3760/cma.j.issn.0253-2727.2021.10.004

**Published:** 2021-10

**Authors:** 海啸 张, 爱明 庞, 欣 陈, 荣莉 张, 卫华 翟, 巧玲 马, 栋林 杨, 嘉璘 魏, 祎 何, 四洲 冯, 明哲 韩, 尔烈 姜

**Affiliations:** 中国医学科学院、北京协和医学院血液病医院（中国医学科学院血液学研究所），实验血液学国家重点实验室，国家血液系统疾病临床医学研究中心，天津 300020 State Key Laboratory of Experimental Hematology, National Clinical Research Center for Blood Diseases, Institute of Hematology & Blood Diseases Hospital, Chinese Academy of Medical Sciences & Peking Union Medical College, Tianjin 300020, China

**Keywords:** 急性髓系白血病伴骨髓增生异常相关改变, 造血干细胞移植, 基因突变, Acute myeloid leukemia with myelodysplastic related changes, Hematopoietic stem cell transplantation, Genetic mutations

## Abstract

**目的:**

评价异基因造血干细胞移植（allo-HSCT）治疗急性髓系白血病伴骨髓增生异常相关改变（AML-MRC）的疗效及预后因素，分析AML-MRC患者基因突变谱系并探讨影响移植预后的分子生物学特征。

**方法:**

对2006年至2020年于中国医学科学院血液病医院接受allo-HSCT的75例AML-MRC患者进行回顾性分析。将75例患者分为：H组［既往有骨髓增生异常综合征（MDS）或MDS/骨髓增殖性肿瘤（MPN）病史］、C组（新诊断的AML-MRC伴MDS相关细胞遗传学异常）和M组（新诊断的AML-MRC伴多系发育异常），分析三组患者临床特征差异以及对移植预后的影响。对43例患者行骨髓靶向二代测序（137个基因）。

**结果:**

①75例AML-MRC患者，男41例，女34例，中位年龄41（18～56）岁，中位随访时间为35（95％ *CI* 30～49）个月，中位总生存（OS）时间为78个月（95％*CI* 23个月～未达到）。移植后3年OS率为57.1％（95％*CI* 45.6％～71.4％），无事件生存（EFS）率为52.0％（95％*CI* 40.8％～66.1％），累积复发率（CIR）为26.8％（95％*CI* 16.6％～30.0％），移植相关死亡率（TRM）为22.7％（95％ *CI* 13.2％～33.8％）。多因素分析显示，移植前处于非第1次完全缓解（CR1）状态是移植后OS和EFS的独立危险因素。影响OS的独立危险因素还包括−5/5q−染色体核型异常、移植后未发生慢性移植物抗宿主病（慢性GVHD）。②75例患者中H组59例（78.7％），其中20例转化为白血病（转白）前曾接受去甲基化药物治疗；C组9例（12.0％），M组7例（9.3％）。M、H、C组移植后3年OS率分别为71.4％（95％*CI* 44.7％～100.0％）、55.0％（95％*CI* 41.8％～72.5％）、55.6％（95％ *CI* 31.0％～99.7％）（*P*＝0.700），EFS率分别为71.4％（95％*CI* 44.7％～100.0％）、46.5％（95％*CI* 34.0％～63.8％）、55.6％（95％*CI* 31.0％～99.7％）（*P*＝0.600）；原发性AML-MRC与继发性AML-MRC相比，移植后3年OS、EFS率差异无统计学意义［61.9％（95％*CI* 41.9％～91.4％）对55.0％（95％ *CI* 41.8％～72.5％），*P*＝0.600；61.9％（95％*CI* 41.9％～91.4％）对46.5％（95％*CI* 34.0％～63.8％），*P*＝0.400］。转白前接受去甲基化治疗（20例）与未接受去甲基化治疗（39例）患者比较，转白时间差异无统计学意义［195（16～937）d对162（9～3167）d，*P*＝0.804］，且两组患者OS和EFS差异无统计学意义（*P*＝0.400，*P*＝0.700）。③对43例（57.3％）患者骨髓样本行二代基因测序，共发现73个突变类型，检出率最高的是U2AF1（11例，25.6％），其他检出率>10％的突变包括RUNX1（10例，23.3％）、NRAS（10例，23.3％）、ASXL1（6例，14.0％）、PTPN11（5例，11.6％）、TET2（5例，11.6％）。单因素分析显示U2AF1［*P*＝0.875，*HR*＝1.110（95％ *CI* 0.295～4.195）］、RUNX1［*P*＝0.685，*HR*＝0.728（95％ *CI* 0.157～3.375）］、NRAS［*P*＝0.919，*HR*＝0.923（95％*CI* 0.196～4.334）］突变对移植后OS没有影响。

**结论:**

−5/5q−染色体异常、未发生慢性GVHD、移植前非CR1状态是影响AML-MRC患者移植后OS的独立危险因素；MHC亚组分类不是影响移植预后的因素；去甲基化药物治疗可能无助于延缓MDS患者转白以及延长移植后OS期。

## Abstract (en)

**Objective:**

To evaluate the outcomes and prognostic factors of adults with acute myeloid leukemia with myelodysplastic-related changes (AML-MRC) who received allogeneic hematopoietic stem cell transplantation (allo-HSCT). The genetic mutation lineage of patients with AML-MRC and the molecular mutation affecting the transplantation prognosis was discussed.

**Methods:**

The clinical data of 75 patients with AML-MRC who underwent allo-HACT from 2006 to 2020 were retrospectively analyzed for clinical characteristics, survival, relapse-related indicators, and risk factors affecting transplantation prognosis. Additionally, the clinical characteristics and prognosis of multilineage dysplasia (M) group, history of myelodysplastic syndrome (MDS) or myelodysplastic syndrome/myelodysplastic proliferative tumor (MDS/MPN) (H) group, and MDS related cytogenetic abnormalities (C) group were compared. The bone marrow of 43 patients underwent targeting second-generation sequencing (137 genes).

**Results:**

①There were 41 males and 34 females with a median age of 41 (18–56) years, a median follow-up time of 35 (95％ *CI* 30–49) months, and a median survival time (OS) of 78 (95％ *CI* 23–) months. Three-year OS and event-free survival (EFS) were 57.1％ (95％ *CI* 45.6％–71.4％) and 52.0％ (95％ *CI* 40.8％–66.1％). Also, the three-year cumulative recurrence rate (CIR) and transplant-related mortality rate (TRM) were 26.8％ (95％ *CI* 16.6％–30.0％) and 22.7％ (95％ *CI* 13.2％–33.8％), respectively. Furthermore, multivariate analysis revealed that pre-transplant non-CR1status was an independent risk factor for OS and EFS. Other independent risk factors for OS included abnormal karyotype of −5/5q− chromosome and the absence of chronic graft-versus-host disease (cGVHD) after transplantation. ②Among the 75 patients, 59 (78.7％) were in group H, 20 had received demethylation drugs before turning to AML and nine cases (12.0％) in group C and seven cases (9.3％) in group M. There was no significant difference in the three-year OS and EFS among the three groups [group M *vs* H *vs* C: OS: 71.4％ (95％ *CI* 44.7％–100.0％) *vs* 55.0％ (95％ *CI* 41.8％–72.5％) *vs* 55.6％ (95％ *CI* 31.0％–99.7％), *P*＝0.700; EFS: 71.4％ (95％ *CI* 44.7％–100.0％) *vs* 46.5％ (95％ *CI* 34.0％–63.8％) *vs* 55.6％ (95％ *CI* 31.0％–99.7％), *P*＝0.600]. Compared with primary and secondary AML-MRC, there was no statistically significant difference in the three-year OS and EFS [61.9％ (95％ *CI* 41.9％–91.4％) *vs* 55.0％ (95％ *CI* 41.8％–72.5％), *P*＝0.600; 61.9％ (95％ *CI* 41.9％–91.4％) *vs* 46.5％ (95％ *CI* 34.0％–63.8％), *P*＝0.400]. Furthermore, there was no significant difference in the time to AML between patients who received demethylation treatment before (20 cases) and those who did not (39 cases) [195 (16–937) d *vs* 162 (9–3167) d, *P*＝0.804]. Moreover, there were no statistically significant differences in the three-year OS and EFS between the two groups (*P*=0.400, *P*=0.700). ③ NGS test was performed on bone marrow samples of 43 patients (57.3％), and 73 mutation types were found. Additionally, U2AF1 had the highest mutation incidence (11 cases, 25.6％), and more than 10％ were found: RUNX1 (ten cases, 23.3％), NRAS (ten cases, 23.3％), ASXL1 (six cases, 14.0％), PTPN11 (five cases, 11.6％), TET2 (five cases, 11.6％). Univariate analysis showed U2AF1 [*P*＝0.875, *HR*＝1.110 (95％ *CI* 0.295–4.195)], RUNX1 [*P*＝0.685, *HR*＝0.728 (95％ *CI* 0.157–3.375)], NRAS [*P*＝0.919, *HR*＝0.923 (95％ *CI* 0.196–4.334)] mutation did not affect OS.

**Conclusion:**

Chromosome abnormality of −5/5q−, cGVHD, and non-CR1 status before transplantation were independent risk factors for OS in patients with allo-HSCT and AML-MRC. Additionally, the MHC subgroup classification was not a factor affecting the prognosis of transplantation. Treatment with demethylated drugs may not delay MDS turning to AML and prolong the OS after transplantation.

急性髓系白血病（AML）是一组异质性疾病，是造血前体细胞突变导致原始细胞异常增殖和分化阻滞，从而抑制骨髓正常造血，其病情发展迅速，自然病程仅数月[1]。急性髓系白血病伴骨髓增生异常相关改变（AML-MRC）为WHO造血与髓系肿瘤分类（2008版）[2]率先提出，指外周血或骨髓原始细胞≥20％、伴有骨髓增生异常形态学特征，或此前有骨髓增生异常综合征（MDS）或骨髓增生异常综合征/骨髓增殖性肿瘤（MDS/MPN）病史，或有MDS相关细胞遗传学异常且没有AML伴重现性细胞遗传学异常独立的一类，占AML的25％～34％。与其他类型AML相比，AML-MRC患者对化疗反应差、生存期短，中位生存期仅为5～10个月[3]。

化疗方案不断优化及新型靶向药物及细胞免疫治疗的应用在一定程度改善了AML患者的预后，但异基因造血干细胞移植（allo-HSCT）仍是目前治愈AML的唯一潜在手段。欧洲白血病网（ELN）指南（2017）及国内相关指南均推荐中、高危AML患者首选allo-HSCT[4]–[5]。本研究分析了我中心75例接受allo-HSCT治疗的AML-MRC患者的临床特征、移植结果以及影响移植结果的预后因素，对AML-MRC患者骨髓样本进行了二代测序（NGS）检测（包含137个基因），分析AML-MRC的基因突变谱系。

## 病例与方法

1. 病例来源：选取2006年至2020年于我中心行allo-HSCT的AML-MRC患者75例及其正常供者75例。

所有患者根据细胞形态学、免疫学、细胞遗传学、分子生物学（MICM）分型标准确诊。所有患者符合WHO 2016诊断标准[6]。缺少确诊时血常规、骨髓形态学以及移植时相关资料的患者排除。所有患者入院时均签署临床资料用于医学研究知情同意书。患者移植时年龄均≥18岁。

2. 临床数据收集方法：所有患者及其正常供者临床资料均来源于中国医学科学院血液病医院住院/门诊病历、医渡云及电话随访。患者移植后定期或病情变化时复查骨髓形态学、多参数流式细胞术检测微小残留病（MRD）及个体化监测分子生物学及细胞遗传学异常等。

3. 供者情况：供者包括人类白细胞抗原（HLA）全相合同胞供者和替代供者。替代供者包括HLA不全相合供者、单倍型供者、HLA全相合无关供者、HLA不全相合无关供者。

4. 预处理方案：均采用清髓性预处理（MAC）方案。以白消安（Bu）+环磷酰胺（Cy）+氟达拉滨（Flu）+阿糖胞苷（Ara-C）/去甲氧柔红霉素（IDA）为基础的改良预处理方案为主。部分患者在此基础上加用IDA或（和）地西他滨（DAC）。单倍型移植、无关供者移植在此基础上加用抗胸腺细胞球蛋白（ATG）。

5. 移植物抗宿主病（GVHD）的防治：采用环孢素A（CsA）或他克莫司（FK506）联合短疗程甲氨蝶呤（MTX），包含或不包含霉酚酸酯（MMF）预防GVHD。GVHD的一线治疗选用糖皮质激素，二线治疗选择包括间充质干细胞（MSC）、抗CD25单抗、ATG、依那西普、芦可替尼等。

6. 二代测序：本研究大部分患者检测了如下137个基因突变：ABL1、ANKRD206、ARID1A、ASXL1、ASXL2、ATG2B、ATM、B2M、BCL2、BCL6、BCOR、BCORL1、BIRC3、BRAF、BRINP3、BTK、CALR、CARD11、CASP8、CBL、CCND1、CCND2、CCND3、CCR4、CD28、CD58、CD79B、CDC25C、CDKN1B、CDKN2A、CEBPA、CNOT3、CREBBP、CRLF2、CSF3R、CSNK1A1、CUX1、CXCR4、DDX3X、DDX41、DIS3、DNM2、DNMT3A、DNMT3B、EED、EGR1、EP300、ETNK1、ETV6、EAH2、FAM46C、FAT1、FBXW7、FGFR3、FLT3、GATA1、GNA13、ID3、IDH1、IDH2、IKZF1、IL7R、IRF4、JAK1、JAK2、JAK3、KDM6A、KIT、KLF2、KMT2A、KMT2D、KRAS、MAP2K1、MAPK1、MAX、MED12、MEF2B、MPL、MYC、MYD88、NF1、NOTCH1、NOTCH2、NMP1、NRAS、NT5C2、PAX5、PDGFRB、PHF6、PIGA、PLCG1、PLCG2、PPM1D、PRDM1、PRKCB、PRPS1、PTEN、PTPN11、RAD21、RBBP6、RELN、RHOA、RPL10、RUNX1、SETBP1、SETD2、SF1、SF3B1、SH2B3、SMC1A、SMC3、SPEN、SRP72、SRSF2、STAG2、STAG3、STAT3、STAT5B、SUZ12、TAL1、TCF3、TERT、TET2、TNFAIP3、TNFRSF14、TP53、TPMT、TRAF3、U2AF1、USP7、WHSC1、WT1、XPO1、ZBTB7A、ZMYM3、ZRSR2。

检测方法：①采取送检标本中基因组DNA，分析至少137个基因蛋白质编码区域的点突变和段片段插入/缺失突变。②采取超高多重PCR外显子富集技术进行高通量基因测序，平均测序深度为800×，使用Ion Reporter系统和Variant Reporter软件进行突变分析；利用dbSNP、1000 Genomes、Polyphen-2和COSMIC等权威数据库进行变异注释和氨基酸突变分析。本方法对5％变异频率的突变位点检测率为97％～98％。③FLI3-ITD同时采用PCR方法检测；NPM1-Exon12、CEBPA-TAD、CEB-PABZIP、CALR-EXON9、MPL-Exon10同时采用Sanger测序方法检测。

7. 研究终点和定义：研究终点包括总生存（OS）、无事件生存（EFS）、复发和移植相关死亡（TRM）。OS时间：造血干细胞回输至死亡或末次随访的时间；EFS时间：造血干细胞回输至复发、死亡或末次随访的时间；复发：完全缓解（CR）后外周血出现白血病细胞或骨髓中原始细胞≥5％或出现新的病态造血或髓外白血病细胞浸润；CR1：诱导化疗后首次达到骨髓无白血病状态；粒细胞植入：外周血中性粒细胞计数（ANC）>0.5×10^9^/L连续3 d；血小板植入：外周血小板计数（PLT）>20×10^9^/L连续7 d且脱离血小板输注；原发性AML-MRC：无MDS、MDS/MPN病史，根据细胞遗传学以及病态造血确诊的AML-MRC；继发性AML-MRC：有明确MDS、MDS/MPN病史的AML-MRC。

8. 统计学处理：采用RStudio软件进行数据分析。计量资料采用Mann-Whitney检验；计数资料采用chi-square检验或Fisher's exact检验。OS和EFS采用Kaplan-Meier法，组间比较采用Log-rank检验。Reverse Kaplan-Meier法计算中位随访时间。累积复发率（CIR）和TRM采用竞争性分析，组间比较采用Gray's检验。连续性变量采用受试者工作特性曲线（ROC）方法、平均值或中位数值确定影响生存和复发的cut-off值。影响生存及复发的危险因素分析：单因素分析*P*<0.1的因素纳入Cox回归模型进行多因素分析。双侧检验*P*<0.05定义为差异具有统计学意义。

## 结果

1. 临床资料：75例AML-MRC患者，男41例，女34例，中位年龄41（18～56）岁。FAB分型：M_2_ 10例（13.3％），M_4_ 6例（8.0％），M_5_ 41例（54.7％），M_6_ 4例（5.3％）、不能分类14例（18.7％）。细胞遗传学：复杂核型（CK）者14例，单体核型（MK）4例，+810例，−5/5q− 6例，−7/7q− 5例。75例患者中61例可进行ELN危险度分层，其中预后良好组7例（9.3％），预后中等组24例（32.0％），预后不良组30例（40.0％），但三组OS差异无统计学意义（*P*＝0.400）。首次诱导化疗后获得骨髓形态学无白血病状态（CR1）者36例（48.0％），转化为白血病（转白）后直接移植患者14例（18.7％）。75例AML-MRC患者的临床特征见表1。

**表1:** 75例AML-MRC患者临床特征

项目	H组（59例）	C组（9例）	M组（7例）	*P*值
年龄（岁，*x*±*s*）	42.22±9.15	36.22±12.10	34.29±10.09	0.043
FAB亚型［例（％）］				0.001
AML不能分类	13（22.0）	0（0）	1（14.3）	
M_2_	4（6.8）	5（55.6）	1（14.3）	
M_4_	5（8.5）	1（11.1）	0（0.0）	
M_5_	35（59.3）	3（33.3）	3（42.9）	
M_6_	2（3.4）	0（0）	2（28.6）	
病态造血［例（％）］	17（28.8）	1（11.1）	7（100.0）	<0.001
MF≥2级［例（％）］	7（11.9）	2（22.2）	1（14.3）	0.694
WBC（×10^9^/L，*x*±*s*）	16.41±43.38	29.36±43.35	16.78±28.75	0.693
HGB（g/L，*x*±*s*）	80.59±24.84	84.56±29.10	73.86±18.59	0.693
PLT（×10^9^/L，*x*±*s*）	71.83±61.29	43.11±47.63	173.86±181.32	0.003
LDH（U/L，*x*±*s*）	413.32±375.57	317.15±228.52	437.63±356.10	0.349
骨髓原始细胞比例（*x*±*s*）	0.350±0.140	0.410±0.170	0.380±0.210	0.471
染色体+8异常［例（％）］	11（18.6）	0（0）	0（0）	0.174
CK［例（％）］	4（6.8）	3（33.3）	0（0）	0.026
ELN危险度分层［例（％）］				0.096
预后良好	7（11.9）	0（0）	0（0）	
预后中等	19（32.2）	2（22.2）	3（42.9）	
预后不良	22（37.3）	7（77.8）	1（14.3）	
NA	11（18.6）	0（0.0）	3（42.9）	
移植前状态［例（％）］				0.165
CR1	25（42.4）	7（77.8）	4（57.1）	
未达CR1	20（33.9）	2（22.2）	3（42.9）	
直接移植	14（23.7）	0（0）	0（0）	
ECOG评分>1分［例（％）］	20（33.9）	2（22.2）	1（14.3）	0.478
HCT-CI评分>1分［例（％）］	9（15.3）	0（0）	0（0）	0.250
预处理方案［例（％）］				
含IDA/DAC方案	42（71.2）	3（33.3）	4（57.1）	0.075
其他	17（28.8）	6（66.7）	3（42.9）	
GVHD预防［例（％）］				0.949
CsA	9（15.3）	2（22.2）	2（28.6）	
FK506	19（32.2）	3（33.3）	1（14.3）	
CSA+MMF	9（15.3）	1（11.1）	1（14.3）	
FK506+MMF	22（37.3）	3（33.3）	3（42.9）	
供受者血型相合［例（％）］				0.122
相合	19（32.2）	6（66.7）	2（28.6）	
主/次不合	40（67.8）	3（33.3）	5（71.4）	
供受者性别组合［例（％）］				0.934
女供男	10（16.9）	1（11.1）	1（14.3）	
男供女	17（28.8）	2（22.2）	3（42.9）	
男供男	22（37.3）	5（55.6）	2（28.6）	
女供女	10（16.9）	1（11.1）	1（14.3）	
供者类型［例（％）］				0.636
同胞全相合供者	28（47.5）	4（44.4）	2（28.6）	
替代供者	31（52.5）	5（55.6）	5（71.4）	
慢性GVHD［例（％）］	20（33.9）	3（33.3）	2（28.6）	0.961
Ⅲ/Ⅳ级急性GVHD［例（％）］ CD34^+^细胞（×10^6^/kg，*x*±*s*）	16（27.1） 3.29±1.30	0（0） 3.36±1.13	2（28.6） 3.21±1.10	0.198 0.974
供者年龄（岁，*x*±*s*）	38.10±11.66	38.44±10.04	36.57±12.57	0.94
移植前病程（d，*x*±*s*）	119.66±110.65	269.33±118.66	204.00±90.32	0.001

注：AML-MRC：急性髓系白血病伴骨髓增生异常相关改变；H组：既往有骨髓增生异常综合征（MDS）或MDS/骨髓增殖性肿瘤（MPN）病史；C组：新诊断的AML-MRC伴MDS相关细胞遗传学异常；M组：新诊断的AML-MRC伴多系发育异常；FAB：英法美协作组；AML：急性髓系白血病；M_2_：急性粒细胞白血病成熟型；M_4_：急性粒-单核细胞白血病；M_5_：急性单核细胞白血病；M_6_：红白血病；LDH：乳酸脱氢酶；MF：骨髓纤维化；CK：复杂核型；ELN：欧洲白血病网；CR1：首次诱导患者骨髓性形态学无白血病状态；直接移植组：转化为白血病后直接接受造血干细胞移植；HCT-CI：造血干细胞移植合并症指数；DAC：地西他滨；IDA：去甲氧柔红霉素；CsA：环孢素A；FK 506：他克莫司；MMF：霉酚酸酯；GVHD：移植物抗宿主病；MNC：单个核细胞

2. 复发和生存：随访至2021年2月28日，中位随访时间为17.8（10.0～180.0）个月，确诊至移植的中位时间为133（14～798）d。75例移植的AML-MRC患者中18例（24.0％）复发，30例（40.0％）死亡，其中8例（26.7％）死于复发，中位生存时间为78个月（95％ *CI* 23个月～未达到）。移植后3年OS率为57.1％（95％ *CI* 45.6％～71.4％），EFS率为52.0％（95％ *CI* 40.8％～66.1％）；5年OS率为50.9％（95％*CI* 38.7％～67％），EFS率为49.1％（95％*CI* 37.6％～64.0％）（图1）。3年CIR为26.8％（95％*CI* 16.6％～30.0％）（图2A），TRM为22.7％（95％ *CI* 13.2％～33.8％）（图2B）。

**图1:**
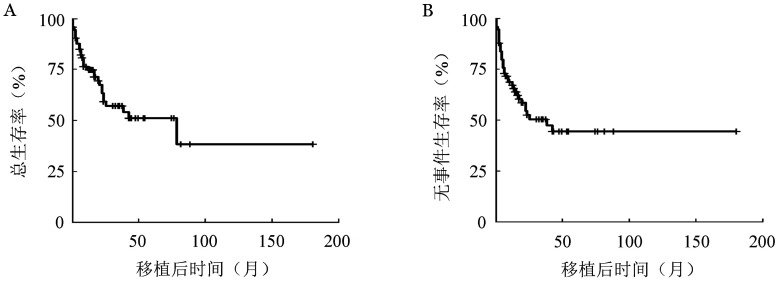
75例急性髓系白血病伴骨髓增生异常相关改变患者异基因造血干细胞移植后总生存曲线（A）和无事件生存曲线（B）

**图2:**
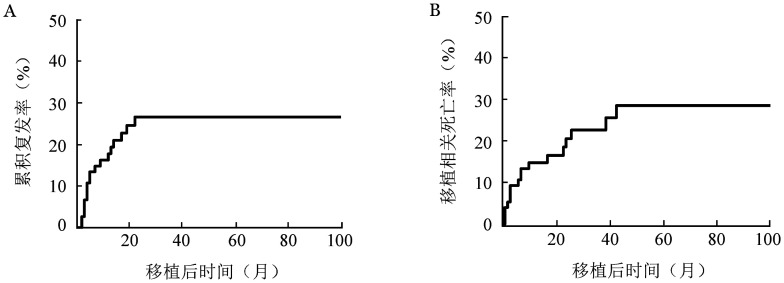
75例急性髓系白血病伴骨髓增生异常相关改变患者异基因造血干细胞移植后累积复发曲线（A）和移植相关死亡曲线（B）

将死亡或复发作为终点，根据ROC曲线、平均值或中位数值确定患者确诊时连续变量的cut-off值：年龄50岁，骨髓原始细胞比例40％，供者年龄35岁，CD34^+^细胞4×10^6^/kg。将患者年龄、AML-MRC亚型、确诊时骨髓原始细胞比例、病态造血、骨髓纤维化（MF）、染色体核型、移植前状态、ECOG评分、HCT-CI评分、预处理方案、供受者ABO血型相合、GVHD预防方案、供受者性别相合、供者类型、慢性GVHD、急性GVHD、供者年龄、CD34^+^细胞计数等纳入单因素分析，分析结果显示：−7/7q−染色体核型异常、−5/5q−染色体核型异常、移植前非CR1状态是OS和EFS共同的预后不良危险因素。直接移植组、CR1组、非CR1组移植后3年OS率分别为43.4％（95％ *CI* 20.5％～92.0％）、74.3％（95％ *CI* 60.0％～92.1％）、41.2％（95％*CI* 24.5％～69.2％）（*P*＝0.010），EFS率分别为37.3％（95％*CI* 16.5％～84.5％）、64.3％（95％ *CI* 49.7％～83.2％）、36.4％（95％*CI* 19.9％～66.5％）（*P*＝0.100）。−7/7q−组、无−7/7q−组移植后3年OS率分别为22.2％（95％*CI* 4.1％～100.0％）、59.8％（95％ *CI* 47.9％～74.6％）（*P*＝0.008），EFS率分别为22.2％（95％*CI* 4.1％～100.0％）、52.5％（95％ *CI* 40.9％～67.4％）（*P*＝0.030）。−5/5q−组、无−5/5q−组移植后3年OS率分别为25.0％（95％*CI* 5.1％～100.0％）、60.3％（95％*CI* 48.5％～75.0％）（*P*＝0.020），EFS率分别为25.0％（95％ *CI* 5.1％～100.0％）、53.1％（95％ *CI* 41.5％～67.8％）（*P*＝0.070）。发生慢性GVHD、未发生慢性GVHD组移植后3年OS率分别为75.8％（95％*CI* 59.3％～96.9％）、47.4％（95％*CI* 33.7％～66.8％）（*P*＝0.030），EFS率分别为63.4％（95％*CI* 47.0％～88.0％）、43.8％（95％*CI* 30.7％～62.4％）（*P*＝0.060）。多因素分析显示，影响OS的独立危险因素包括−5/5q−染色体核型异常、移植前非CR1状态、移植后未发生慢性GVHD。影响EFS的独立危险因素为移植前非CR1状态。单因素和多因素分析结果详见表2。

**表2:** 影响急性髓系白血病伴骨髓增生异常相关改变患者总生存（OS）和无事件生存（EFS）危险因素分析

因素	OS单因素分析		OS多因素分析		EFS单因素分析		EFS多因素分析	
	*P*值	*HR*（95％*CI*）	*P*值	*HR*（95％*CI*）	*P*值	*HR*（95％*CI*）	*P*值	*HR*（95％*CI*）
移植前状态	0.008	1.861（1.179～2.937）	0.001	2.255（1.383～3.676）	0.032	1.577（1.041～2.388）	0.025	1.621（1.062～2.473）
慢性GVHD	0.032	0.391（0.166～0.923）	0.016	0.324（0.130～0.808）	0.140	0.572（0.272～1.202）	0.136	0.550（0.251～1.206）
−5/5q−	0.026	3.373（1.158～9.824）	0.035	3.638（1.096～12.080）	0.065	2.700（0.940～7.700）	0.154	2.457（0.715～8.444）
−7/7q−	0.012	3.944（1.348～12.537）	0.313	1.903（0.546～6.635）	0.015	3.777（1.300～10.981）	0.166	2.546（0.678～9.557）
骨髓原始细胞比例					0.096	0.492（0.213～1.344）	0.124	0.510（0.216～1.202）

注：移植前状态包括移植前未接受化疗、诱导化疗后首次达到骨髓无白血病状态、诱导化疗后骨髓白血病细胞>5％或首次达到骨髓无白血病状态后复发；GVHD：移植物抗宿主病

3. 亚组分析：根据WHO 2016诊断标准，将75例AML-MRC患者分为三组：H组（既往有MDS或MDS/MPN病史）、C组（新诊断的AML-MRC伴MDS相关细胞遗传学异常）和M组（新诊断的AML-MRC伴多系发育异常）。75例患者中H组患者59例（78.7％），其中转白前曾接受去甲基化药物治疗20例。H组59例患者自诊断MDS、MDS/MPN至转白的中位时间为243（9～3167）d。转白前接受去甲基化治疗（20例）和未接受去甲基化治疗（39例）患者的转白时间分别为195（16～937）d、162（9～3167）d（*P*＝0.804），两组3年OS率分别为42.3％（95％ *CI* 20.8％～86.2％）、59.3％（95％ *CI* 44.5％～79.0％）（*P*＝0.400），EFS率分别为38.3％（95％*CI* 18.7％～78.4％）、50.6％（95％*CI* 36.2％～70.6％）（*P*＝0.700）。C组患者9例（12％），其细胞遗传学异常组成比例为：−5/5q−者4例，−7/7q−者3例，其中同时伴−5/5q−、−7/7q−者1例，del（11q）者2例，CK者1例，MK者0例。M组患者7例（9.3％）。三组临床特征比较结果见表1。三组间年龄（*P*＝0.043）、FAB亚型（*P*＝0.001）、血小板计数（*P*＝0.003）差异有统计学意义，其中H组年龄显著高于C组（*P*＝0.046），C组血小板计数显著低于M组（*P*＝0.045）。生存分析显示，M、H、C组移植后3年OS、EFS差异无统计学意义［OS：71.4％（95％*CI* 44.7％～100.0％）对55.0％（95％ *CI* 41.8％～72.5％）对55.6％（95％ *CI* 31.0％～99.7％），*P*＝0.700；EFS：71.4％（95％ *CI* 44.7％～100.0％）对46.5％（95％*CI* 34.0％～63.8％）对55.6％（95％*CI* 31.0％～99.7％），*P*＝0.600］（图3），原发性AMLMRC组与继发性AML-MRC组移植后3年OS、EFS差异无统计学意义［OS：61.9％（95％*CI* 41.9％～91.4％）对55.0％（95％ *CI* 41.8％～72.5％），*P*＝0.600；EFS：61.9％（95％ *CI* 41.9％～91.4％）对46.5％（95％*CI* 34.0％～63.8％），*P*＝0.400］。

**图3:**
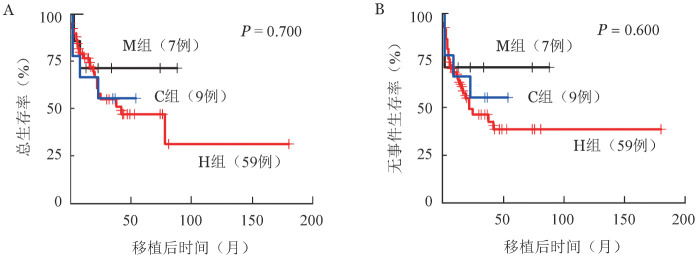
不同亚组急性髓系白血病伴骨髓增生异常相关改变（AML-MRC）患者异基因造血干细胞移植后总生存（A）和无事件生存曲线（B） H组：转化为白血病前有骨髓增生异常综合征（MDS）或MDS/骨髓增殖性肿瘤（MPN）病史；C组：伴MDS相关细胞遗传学异常；M组：伴多系发育异常

4. 43例AML-MRC患者基因突变分析：75例患者中43例（57.3％）患者骨髓样本行NGS检测，其中H组36例（83.7％），C组3例（7.0％），M组4例（9.3％）。43例患者骨髓样本中共发现73个突变类型，人均检出1.7个突变。检出率最高的突变是U2AF1（11例，25.6％），检出率>10％的突变包括RUNX1（10例，23.3％）、NRAS（10例，23.3％）、ASXL1（6例，14.0％）、PTPN11（5例，11.6％）、TET2（5例，11.6％）。此外，NMP1突变者4例（9.3％），均基于继发性AML-MRC诊断；FLT3突变者3例（7.0％），其中FLT3-ITD 1例（2.3％）。39例（90.7％）患者发生2个及以上突变，最多同时发生8个基因突变，3例（7.0％）患者发生1个基因突变，1例（2.3％）患者未检出任何突变。43例AML-MRC患者基因突变谱系见图4。

**图4:**
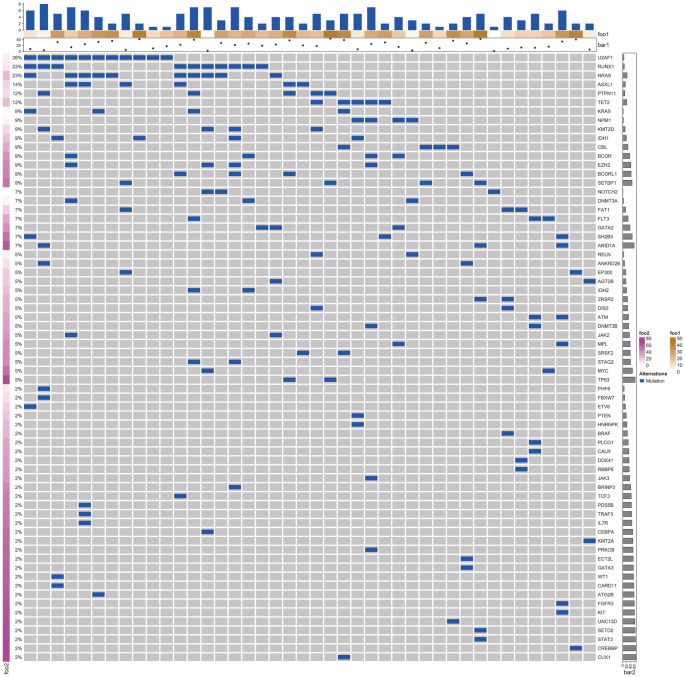
43例急性髓系白血病伴骨髓增生异常相关改变患者基因突变谱系

我们将可能影响预后的临床因素及基因突变纳入预后分析，其中基因突变选择突变例数较多的的U2AF1、RUNX1、NRAS突变。单因素分析显示U2AF1 ［*P*＝0.875，*HR*＝1.110 （95％ *CI* 0.295～4.195）］、RUNX1［*P*＝0.685，*HR*＝0.728（95％ *CI* 0.157～3.375）］、NRAS［*P*＝0.919，*HR*＝0.923（95％*CI* 0.196～4.334）］不是影响移植OS的因素。

## 讨论

AML-MRC是一组异质性疾病，其临床特征包括MDS、MDS/MPN病史或伴有MDS相关细胞遗传学异常或伴有多系发育异常，随着对疾病认识的增加，WHO 2016对AML-MRC的诊断在细节上做了进一步修订[6]，在临床工作中，AML-MRC的诊断具有一定的难度。AML-MRC与非AML-MRC相比，传统化疗反应差，生存期短，其原因可能是AML-MRC拥有独特的临床和生物学特征。因此，建议达到CR且有合适供者的患者行allo-HSCT[7]。

近年来，已有研究证实AML-MRC的临床特征可能是化疗的不良预后因素[3]，但是否影响移植结果尚不完全明确。一项日本的全国性研究显示，4 091例AML-MRC患者移植后3年OS率为35.5％，3年CIR为32.1％，病态造血与移植后较长的OS相关，而有MDS、MDS/MPN病史与移植预后无关[8]。本研究中纳入75例AML-MRC移植患者，是国内报道病例数最多的单中心研究，随访时间长达15年，移植后3年OS、CIR分别为57.1％（95％*CI* 45.6％～71.4％）、26.8％（95％*CI* 16.6％～30.0％），结果优于上述报道。多因素分析显示，影响移植OS的独立预后因素包括−5/5q−染色体异常、移植前状态和移植后慢性GVHD。AML-MRC亚组分类（M、H、C组）与移植预后无关，即使三组年龄和血小板计数差异具有统计学意义（*P*＝0.043，*P*＝0.003）。一项纳入147例AML移植患者的单中心研究[9]与本研究结果相似，该研究结果显示AML-MRC不是影响移植结果的独立预后因素；AML-MRC患者与AML-NOS患者的2年OS、CIR和非复发死亡（NRM）差异均不具有统计学意义（OS：48％对59％；CIR：37％对35％；NRM：19％对13％）。此外，本研究还发现，既往有MDS、MDS/MPN病史患者转白前是否接受去甲基化药物治疗与移植预后无关。AML-MRC亚型即MDS、MDS/MPN病史、MDS相关细胞遗传学异常和多系发育异常不是影响移植预后的因素，可能有两方面的原因，一方面，allo-HSCT能够克服AML-MRC不良的临床特征。另一方面，可能是由于各亚组具有相似的基因突变谱系，相似的生物学特性决定各亚组实质相同。

虽然本研究显示MDS相关细胞遗传学这一亚组不是影响移植预后的独立因素，但−5/5q−是影响移植OS的独立危险因素［*P*＝0.026，*HR*＝3.4（95％*CI* 1.2～9.8）］，与文献[8]报道一致。此外，−7/7q−、CK、MK染色体异常不是影响移植OS的独立危险因素，与ELN指南[4]不一致。但生存分析显示−7/7q−组和无−7/7q−组3年OS和EFS率均具有统计学差异（OS：22.2％对59.8％，*P*＝0.008；EFS：22.2％对52.5％，*P*＝0.03），因此−7/7q−染色体异常对移植预后的影响需要进一步研究。本研究中69例AML-MRC患者可行ELN遗传学分层，低、中、高危组移植后3年OS率差异无统计学意义（*P*＝0.400）。以上结果均显示，对于AML-MRC这类疾病而言，ELN危险分层可能并不能满足移植预后评估的需求，新的、特异性的AML-MRC预后分层标准亟需进一步探索。Harada等[8]提出一个用于评估移植预后的分层标准，低危组因素包括：病态造血、MDS或MDS/MPN病史、−7/7q−和其他色体异常（1088例，64.5％），中危组因素包括CK和−5/5q−（273例，16.2％），高危组因素包括MK（326例，19.3％），低、中、高危组3年OS率分别为50.7％、36.9％、13.8％（*P*<0.001）。此外，我们也应该在分子生物学方面做更多的工作以用于该类疾病诊断和预后评估。

随着高通量测序技术的发展，AML的分子突变谱系趋于清晰[10]–[17]。Lindsley等[18]报道，SRSF2、SF3B1、U2AF1、ZRSR2、ASXL1、EZH2、BCOR和STAG2突变对于继发性AML的诊断具有95％的特异性，与本组患者的基因突变谱系高度重合。本研究分析结果显示突变病例数较多的突变（U2AF1、RUNX1、NRAS）均与移植结果无关，这可能是由于本研究行二代测序病例数较少（43例），而测序的基因数目多达137个，不利于发现影响移植结果的突变。但既往其他研究报道了不同基因突变对AMLMRC患者移植疗效的影响。Devillier等[19]报道AML-MRC患者伴有ASXL1高频率突变和NPM1、FLT3、DNMT3A低频率突变，且ASXL1和TP53突变是AML-MRC预后不良的独立危险因素[20]。Tetsuichi等[13]研究显示，对于MDS、MDS/MPN和继发性AML而言，TP53突变阳性或RAS通路相关突变是移植不良预后的危险因素，尤其是TP53合并CK的患者。因此移植治疗策略的制定应基于临床特征、细胞及分子生物学特征。本研究中，H组转白前去甲基化药物治疗并未延长MDS、MDS/MPN患者的转白时间以及移植后OS，可能提示了去甲基化药物并不能克服AML-MRC患者的分子生物学变异。因此识别AML-MRC分子突变谱系的重要意义在于帮助识别哪一类MDS患者有转白倾向，以期尽早指定治疗策略。

综上所述，本研究结果显示−5/5q−染色体异常、慢性GVHD、移植前非CR1状态是影响AML-MRC患者移植后OS的独立危险因素；MHC亚组分类不是影响移植预后的因素；去甲基化药物治疗可能不能延缓MDS患者转白以及延长移植后OS。本组病例涉及的预处理方案种类较多，我们未对不同预处理方案患者进行预后分析。影响AML-MRC移植预后的因素及其分子突变特征需要多中心前瞻性的研究来证实。

## References

[1] Döhner H, Weisdorf DJ, Bloomfield CD (2015). Acute Myeloid Leukemia[J]. N Engl J Med.

[2] Vardiman JW, Thiele J, Arber DA (2009). The 2008 revision of the World Health Organization (WHO) classification of myeloid neoplasms and acute leukemia: rationale and important changes[J]. Blood.

[3] Xu XQ, Wang JM, Gao L (2014). Characteristics of acute myeloid leukemia with myelodysplasia-related changes: A retrospective analysis in a cohort of Chinese patients[J]. Am J Hematol.

[4] Döhner H, Estey E, Grimwade D (2017). Diagnosis and management of AML in adults: 2017 ELN recommendations from an international expert panel[J]. Blood.

[5] 中华医学会血液学分会白血病淋巴瘤学组 (2017). 成人急性髓系白血病(非急性早幼粒细胞白血病)中国诊疗指南(2017年版)[J]. 中华血液学杂志.

[6] Arber DA, Orazi A, Hasserjian R (2016). The 2016 revision to the World Health Organization classification of myeloid neoplasms and acute leukemia[J]. Blood.

[7] Li Z, Labopin M, Ciceri F (2018). Haploidentical transplantation outcomes for secondary acute myeloid leukemia: Acute Leukemia Working Party (ALWP) of the European Society for Blood and Marrow Transplantation (EBMT) study[J]. Am J Hematol.

[8] Harada K, Konuma T, Machida S (2019). Risk stratification and prognosticators of acute myeloid leukemia with myelodysplasia-related changes in patients undergoing allogeneic stem cell transplantation: a retrospective study of the Adult Acute Myeloid Leukemia Working Group of the Japan Society for Hematopoietic Cell Transplantation[J]. Biol Blood Marrow Transplant.

[9] Ikegawa S, Doki N, Kurosawa S (2016). Allogeneic hematopoietic stem cell transplant overcomes poor prognosis of acute myeloid leukemia with myelodysplasia-related changes[J]. Leuk Lymphoma.

[10] Ley TJ, Miller C, Ding L (2013). Genomic and epigenomic landscapes of adult de novo acute myeloid leukemia[J]. N Engl J Med.

[11] Walter MJ, Shen D, Ding L (2012). Clonal architecture of secondary acute myeloid leukemia[J]. N Engl J Med.

[12] Bullinger L, Döhner K, Döhner H (2017). Genomics of acute myeloid leukemia diagnosis and pathways[J]. J Clin Oncol.

[13] Yoshizato T, Nannya Y, Atsuta Y (2017). Genetic abnormalities in myelodysplasia and secondary acute myeloid leukemia: impact on outcome of stem cell transplantation[J]. Blood.

[14] Welch JS, Ley TJ, Link DC (2012). The origin and evolution of mutations in acute myeloid leukemia[J]. Cell.

[15] Ding L, Ley TJ, Larson DE (2012). Clonal evolution in relapsed acute myeloid leukaemia revealed by whole-genome sequencing[J]. Nature.

[16] Patel JP, Gönen M, Figueroa ME (2012). Prognostic relevance of integrated genetic profiling in acute myeloid leukemia[J]. N Engl J Med.

[17] Papaemmanuil E, Gerstung M, Bullinger L (2016). Genomic classification and prognosis in acute myeloid leukemia[J]. N Engl J Med.

[18] Lindsley RC, Mar BG, Mazzola E (2015). Acute myeloid leukemia ontogeny is defined by distinct somatic mutations[J]. Blood.

[19] Devillier R, Gelsi-Boyer V, Brecqueville M (2012). Acute myeloid leukemia with myelodysplasia-related changes are characterized by a specific molecular pattern with high frequency of ASXL1 mutations[J]. Am J Hematol.

[20] Devillier R, Mansat-De Mas V, Gelsi-Boyer V (2015). Role of ASXL1 and TP53 mutations in the molecular classification and prognosis of acute myeloid leukemias with myelodysplasia-related changes[J]. Oncotarget.

